# Long-Term Static Cultivation Alters Lipid Metabolism and Bioenergetic Capacity in A549 Cells

**DOI:** 10.3390/ijms27083417

**Published:** 2026-04-10

**Authors:** Ivana Ďurišová, Lucia Šofranková, Aleš Kvasnička, Miroslav Baláž, Ivana Fábryová, David Friedecký, Mária Balážová

**Affiliations:** 1 Department of Membrane Biochemistry, Institute of Animal Biochemistry and Genetics, Centre of Biosciences, Slovak Academy of Sciences, 84104 Bratislava, Slovakia; 2 Institute of Biochemistry and Microbiology, Faculty of Chemical and Food Technology, Slovak University of Technology, 81237 Bratislava, Slovakia; 3 Department of Medical Biochemistry, Oslo University Hospital, 4950 Oslo, Norway; 4 Laboratory for Inherited Metabolic Disorders, Department of Clinical Biochemistry, University Hospital Olomouc, Faculty of Medicine and Dentistry, Palacký University Olomouc, 77900 Olomouc, Czech Republic; 5 Biomedical Research Center, Slovak Academy of Sciences, 84104 Bratislava, Slovakia; 6 Department of Animal Physiology and Ethology, Faculty of Natural Sciences, Comenius University, 84215 Bratislava, Slovakia

**Keywords:** A549, cells, static culture, lipid metabolism, mitochondrial respiration, phosphatidylglycerol biosynthesis, cellular stress

## Abstract

A549 cells are widely used as an in vitro model of alveolar type II (ATII) epithelial cells; however, their phenotype and metabolic state are highly sensitive to culture conditions, cell density, and the duration of static, non-passaged cultivation. Here, we examined how prolonged static culture affects lipid metabolism, mitochondrial bioenergetics, and viability in A549 cells. A549 cultures were maintained without passaging for up to 25 days in DMEM or Ham’s F-12 and analyzed using lipid secretion assays, targeted lipidomics, [^14^C]-acetate incorporation, Seahorse bioenergetic profiling, and transcriptional analysis of stress-associated markers. Several surfactant-associated readouts were highest during early culture, peaking on day 7, as evidenced by elevated expression of *ABCA3* and SP-A and maximal secretion of surfactant-associated phospholipids. With prolonged cultivation and increasing culture density, cellular phosphatidylglycerol levels declined progressively and became nearly undetectable by day 25, accompanied by reduced anabolic lipid metabolism, lower oxygen consumption, and impaired glycolytic activity. These changes coincided with increased reactive oxygen species, elevated intracellular Ca^2+^ levels, and increased expression of stress-associated transcripts, including *CASP1*, *IL1B*, and *C3*. Later stages were also associated with reduced mitochondrial respiration and decreased viability. Collectively, our findings show that prolonged static culture is associated with metabolic remodeling and reduced bioenergetic capacity in A549 cells.

## 1. Introduction

Pulmonary surfactant is a lipid–protein complex essential for reducing surface tension in the alveoli and for modulating immune responses in the lung [1,2]. Its lipid fraction is dominated by phosphatidylcholine (PC), phosphatidylglycerol (PG), and cholesterol, whereas surfactant proteins contribute to its assembly and host defense functions. The biosynthesis and secretion of surfactant lipids are hallmarks of alveolar type II (ATII) epithelial cells, which also serve as progenitors for alveolar type I (ATI) cells during lung repair [3]. Dysregulation of surfactant metabolism is implicated in a wide spectrum of pulmonary diseases, including acute respiratory distress syndrome, pneumonia, and chronic obstructive pulmonary disease [4].

Human lung adenocarcinoma A549 cells are widely used as an ATII-related epithelial cell model in vitro, because they retain the capacity for phospholipid synthesis and display selected surfactant-associated properties [5,6,7]. However, their malignant origin and long-term adaptation to in vitro cultivation are associated with reduced differentiation capacity and altered regulation of surfactant-related pathways [8,9]. Importantly, epithelial cell phenotype, metabolic activity, and inflammatory signaling are highly sensitive to culture parameters, including medium composition, serum availability, cell density, and the duration of static cultivation, as demonstrated across multiple pulmonary epithelial models [10,11]. Under conventional static culture conditions without passaging, increasing cell density is inevitable over time and induces pericellular hypoxia, metabolic suppression, oxidative stress, and inflammatory signaling in epithelial cultures [12,13,14]. Despite these limitations, A549 cells are frequently maintained under static conditions for extended periods in studies of chronic exposure, inflammation, viral infection, and surfactant-related processes, sometimes up to 21–25 days [9,15,16,17]. In such experimental setups, “culture duration” is inherently coupled to increasing confluence/crowding and the accumulation of stressors in the local microenvironment; therefore, late time points may reflect consequences of sustained over-confluence rather than intrinsic time-dependent phenotypic programming. Consequently, experimental observations obtained at later stages of static cultivation may reflect a combined influence of prolonged culture and progressively increasing cell density. Defining the temporal window during which A549 cells maintain stable surfactant-associated metabolic activity in this culture paradigm is therefore important for interpreting long-term experiments.

A key surfactant lipid is PG, which not only contributes to alveolar stability but also modulates immune responses during microbial infection [18]. PG serves as a precursor for cardiolipin (CL) biosynthesis in mitochondria, thereby providing a biochemical link between surfactant-associated lipid metabolism and mitochondrial lipid homeostasis. While PG depletion has been observed in various models of lung injury and inflammation [19,20], the dynamics of PG synthesis and secretion in A549 cells under prolonged static culture conditions remain poorly characterized, particularly in the context of increasing cell density and metabolic stress. Characterizing these dynamics is important because metabolic stress, mitochondrial dysfunction, and lipid remodeling frequently accompany prolonged high-density culture conditions and may contribute to phenotypic drift in in vitro cell models [21].

Here, we investigated how medium composition and long-term cultivation under progressively increasing culture density inherent to static, non-passaged conditions influence lipid secretion, phospholipid remodeling, mitochondrial PG biosynthesis, and respiratory activity in A549 cells. Using a combination of lipidomic, metabolic, and phenotypic analyses, we assessed how prolonged static culture affects lipid anabolic capacity, mitochondrial bioenergetics, and selected surfactant-associated cellular features. Our data indicate that A549 cultures exhibit the highest levels of several surfactant-associated readouts during early cultivation. In contrast, later stages are characterized by suppressed anabolic metabolism, reduced mitochondrial respiration, and increased stress-associated signatures. Rather than attempting to uncouple the effects of culture duration and cell density, which are intrinsically linked in static, non-passaged systems, the present study aims to characterize how these combined factors shape lipid metabolism and cellular bioenergetics during prolonged cultivation of A549 cells. In this context, our results define an operational temporal window during which static A549 cultures exhibit the most stable lipid metabolic characteristics, whereas later time points predominantly reflect high-density, stress-associated remodeling of cellular metabolism.

## 2. Results

### 2.1. Lipid Metabolism and Mitochondrial Function in A549 Cells Under Long-Term Cultivation

**Figure 1:**
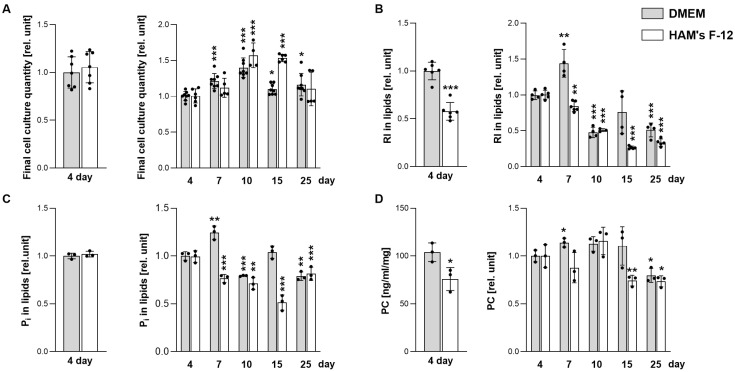
Lipid secretion in A549 cells during long-term cultivation. A549 cells were cultured for 4, 7, 10, 15, and 25 days in low-glucose DMEM or low-glucose Ham’s F-12 medium. (**A**) Total cellular protein per dish as an indicator of culture expansion (cell accumulation). (**B**) Secretion of total lipids into the culture medium, assessed using [^14^C]-acetate labeling. (**C**) Secretion of phospholipids and sphingolipids into the medium, quantified based on inorganic phosphate content (Pi). (**D**) Secretion of phosphatidylcholine (PC) into the medium, measured by mass spectrometry. (**A**–**D**) Data represent mean ± SD from at least three independent experiments (individual points shown). Values were normalized to mL of medium and mg of cellular protein, and additionally expressed relative to 4-day cultures in DMEM (left panels) or to 4-day cultures in the respective medium over time (right panels). Statistically significant differences between media (DMEM vs. Ham’s F-12; left panels) or between prolonged cultures and 4-day cultures (right panels) are indicated: * *p* < 0.05; ** *p* < 0.01; *** *p* < 0.001. RI, radioactivity.

**Figure 2:**
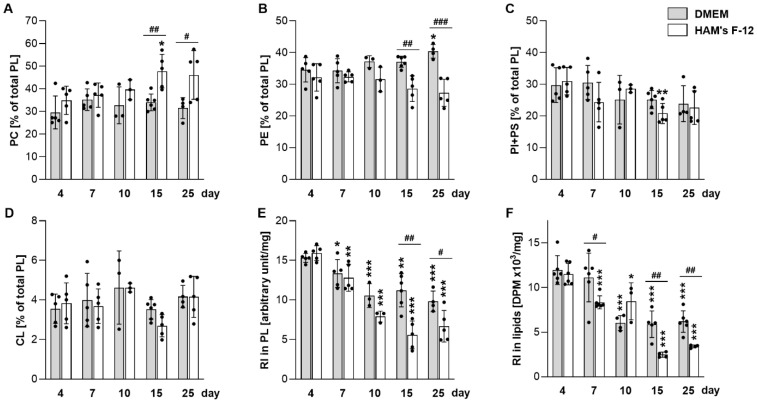
Intracellular phospholipid composition in A549 cells. A549 cells were labeled with [^14^C]-acetate, and lipids extracted from cell homogenates were separated. (**A**–**D**) Relative amounts of individual phospholipids were calculated based on the radioactivity of individual spots. (**E**) Total radioactivity in phospholipids and (**F**) in total cell lipids, normalized to protein content. (**A**–**F**) Data represent mean ± SD from at least three independent experiments (individual points shown). Statistically significant differences between long-term cultures and 4-day cultures (asterisks) or between cells cultured in Ham’s F-12 and DMEM (hashtag) are indicated: * or ^#^ *p* < 0.05; ** or ^##^ *p* < 0.01; *** or ^###^ *p* < 0.001. CL, cardiolipin; DPM, disintegrations per minute; PC, phosphatidylcholine; PE, phosphatidylethanolamine; PI, phosphatidylinositol; PL, phospholipids; PS, phosphatidylserine; RI, radioactivity.

We monitored surfactant-related lipid secretion and intracellular lipid composition in A549 cells over a 25-day cultivation period in two commonly used media, Dulbecco’s Modified Eagle Medium (DMEM) and Ham’s Nutrient Mixture F-12 (Ham’s F-12), to assess the effects of medium composition and prolonged static conditions on lipid metabolism and cell growth (Figure 1 and Figure 2). Total cellular protein per culture (used here as an estimate of attached cellular biomass rather than absolute cell number) increased during the first 10 days in both media, with Ham’s F-12 cultures reaching higher levels by day 15. By day 25, protein levels declined in both groups (Figure 1A), suggesting a reduction in attached cellular biomass under prolonged static cultivation. This pattern is consistent with constraints emerging in long-term high-density epithelial cultures, where increasing confluence can be associated with pericellular hypoxia and broader metabolic limitation [12,14,21].

We subsequently quantified lipid secretion using [^14^C]-acetate labeling. By day 4, DMEM-cultured cells secreted ~42% more radiolabeled lipids than those grown in Ham’s F-12 (Figure 1B). This difference increased by day 7 (+44% in DMEM relative to day 4, whereas secretion decreased by ~16% in Ham’s F-12). Biochemical quantification of total phosphate-containing lipids showed a similar pattern, peaking on day 7 in DMEM cultures, then progressively decreasing over time (Figure 1C). Mass spectrometry further confirmed that PC, the dominant surfactant phospholipid, followed this trend, with secretion highest on day 7 and declining significantly by day 25 (Figure 1D). The fatty acid composition of PC differed between DMEM and Ham’s F-12, with Ham’s F-12 cultures displaying a slight increase in PC 16:0–18:1 and a corresponding decrease in PC 16:0–16:1 and PC 16:0–18:2 relative to DMEM. These differences remained largely stable throughout the cultivation period (Supplementary Figure S1). To further address the effect of changing culture density, we additionally normalized lipid secretion to cell number in DMEM cultures. This alternative normalization supported the same overall pattern as protein normalization, with a clear late-stage decline in secretion into the medium. In particular, secretion of total radiolabeled lipids and phosphate-containing lipids was markedly reduced from day 10 onward, whereas PC secretion showed a significant decline by day 25, consistent with the protein-normalized data (Supplementary Figure S2).

To determine whether differences in lipid secretion reflect altered intracellular synthesis, we analyzed intracellular phospholipids. Cells were labeled with [^14^C]-acetate during the final 48 h of each cultivation period and subsequently harvested for lipid extraction and class distribution analysis (Figure 2). Because labeling was performed for an extended period (48 h), the measured incorporation reflects overall lipid turnover under these conditions rather than instantaneous synthesis rates. The most pronounced medium-dependent differences were observed for PC and phosphatidylethanolamine (PE). In Ham’s F-12, PC levels progressively increased at the expense of PE, most prominently at days 15 and 25 (Figure 2A,B). Incorporation of [^14^C]-acetate into phospholipids and total organic-phase macromolecules was markedly reduced at later time points, particularly in Ham’s F-12 cultures (Figure 2E,F), indicating a broad reduction in anabolic activity at the culture level rather than selective remodeling of individual lipid classes.

**Figure 3:**
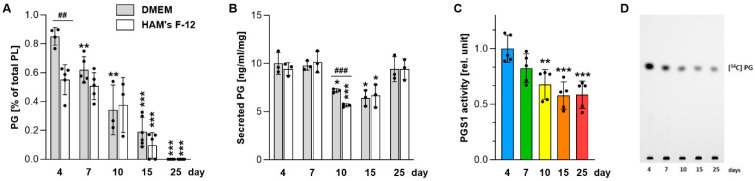
PG biosynthesis and secretion in A549 cells. (**A**) Relative cellular PG levels, quantified from the radioactivity of TLC-separated lipid spots. (**B**) PG secretion into the culture medium, determined by mass spectrometry. (**C**) PGS1 enzyme activity measured in cell homogenates cultured in DMEM. (**D**) Representative TLC image showing radioactive products of the PGS1 assay. Data represent mean ± SD from at least three independent experiments (individual data points shown). Statistically significant differences between long-term cultures and 4-day cultures are indicated by asterisks, and between cells cultured in Ham’s F-12 and DMEM by hashtags are indicated: * *p* < 0.05; ** or ^##^ *p* < 0.01; *** or ^###^ *p* < 0.001. PG, phosphatidylglycerol; PL, total phospholipids.

**Figure 4:**
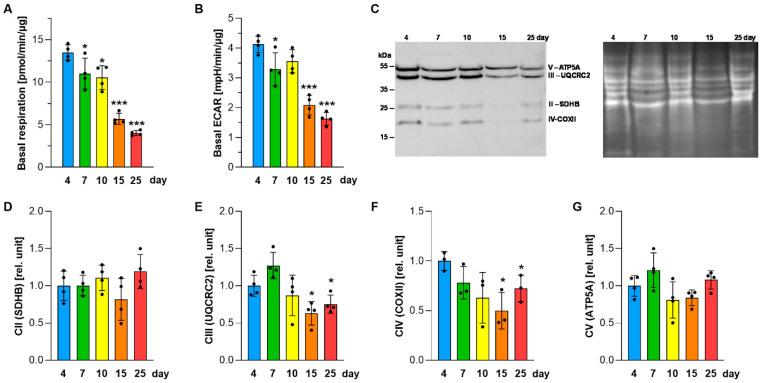
Mitochondrial respiration and glycolytic activity in A549 cells. A549 cells were cultured in DMEM throughout the experiment. (**A**) Basal oxygen consumption rate was measured in intact cells as an indicator of mitochondrial respiration. (**B**) Extracellular acidification rate (ECAR) was determined as an indicator of glycolytic activity. (**C**) Representative immunoblot showing protein levels of mitochondrial respiratory chain subunits SDHB (Complex II, CII), UQCRC2 (Complex III, CIII), COXII (Complex IV, CIV), and ATP5A (ATP synthase, CV) (left), with No-Stain™ total-protein labeling shown for normalization (right). (**D**–**G**) Quantification of individual respiratory complex subunits and ATP synthase levels during the cultivation period, normalized to No-Stain™ total protein per lane. Data represent mean ± SD from at least three independent experiments (individual data points shown). Statistically significant differences between long-term cultures and 4-day cultures are indicated: * *p* < 0.05; *** *p* < 0.001.

Among the analyzed phospholipids, PG exhibited particularly strong time-dependent changes, prompting further analysis of its biosynthesis and functional consequences. PG synthesis declined progressively during prolonged static conditions, becoming nearly undetectable by day 25 in both DMEM and Ham’s F-12 (Figure 3A), whereas CL levels remained largely stable (Figure 2D). PG secretion was also reduced, with more than a 30% decrease at days 10 and 15 compared with 4-day cultures (Figure 3B), accompanied by a progressive decline in PGS1 (phosphatidylglycerophosphate synthase; the key enzyme in de novo PG biosynthesis) activity to approximately 40% of baseline by day 25 (Figure 3C,D). These changes coincided with substantial reductions in mitochondrial bioenergetic parameters. Basal respiration dropped by nearly 70% in 25-day cultures compared with 4-day cultures (Figure 4A), accompanied by reduced basal extracellular acidification rate (ECAR), indicative of suppressed glycolytic activity (Figure 4B). These bioenergetic impairments were further associated with decreased levels of respiratory chain Complex III subunit UQCRC2 and Complex IV subunit COXII (Figure 4C–G and Supplementary Figure S4).

Together, these results show that long-term cultivation of A549 cells is associated with changes in lipid secretion and intracellular de novo phospholipid synthesis, a gradual decline in PG biosynthesis, and impaired mitochondrial respiration. Because static culture inherently leads to progressively increasing cell density, these alterations occur in parallel with the development of high-density culture conditions at later time points.

### 2.2. Phenotypic Changes in A549 Cells During Long-Term Cultivation

**Figure 5:**
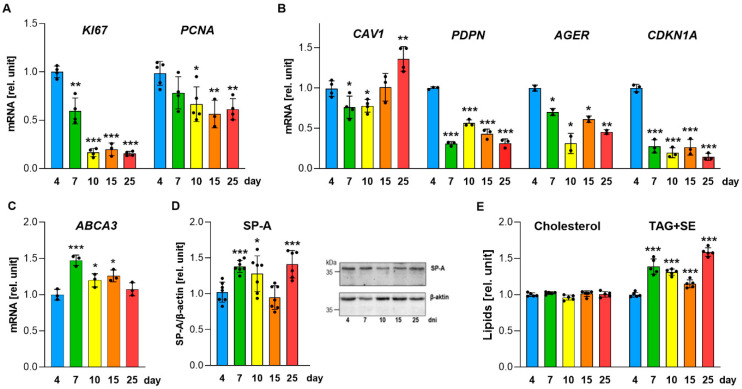
Phenotypic markers and lipid features of A549 cells. A549 cells were cultured in DMEM throughout the experiment. (**A**) Expression of proliferation markers *KI67* and *PCNA* determined by RT-qPCR. (**B**) Expression of phenotypic markers *CAV1*, *PDPN*, *AGER*, and *CDKN1A* determined by RT-qPCR. (**C**) Expression of the ATII marker *ABCA3* determined by RT-qPCR. (**D**) Intracellular SP-A protein levels during 25 days of long-term static cultivation (left) and representative immunoblot analysis of SP-A and β-actin (right), used for normalization. (**E**) Cellular cholesterol and neutral lipid levels (TAG and SE) determined spectrophotometrically at 475 nm after sulfuric acid staining. (**A**–**E**) Data represent mean ± SD from at least two independent experiments (individual points shown) and were normalized to 4-day cultures. Statistically significant differences between long-term cultures and 4-day cultures are indicated: * *p* < 0.05; ** *p* < 0.01; *** *p* < 0.001. SE, sterol esters; SP-A, surfactant protein A; TAG, triacylglycerols.

To assess whether long-term cultivation induces phenotypic changes in A549 cells, we analyzed proliferation and lineage-specific markers (Figure 5). Proliferation markers *KI67* and *PCNA* declined progressively, consistent with reduced proliferative activity under high cell density (Figure 5A). Importantly, downregulation of these markers does not necessarily indicate differentiation, as it may also reflect cellular stress, senescence, or activation of cell death pathways [22,23]. We subsequently examined potential shifts toward ATI-like or alternative epithelial phenotypes by measuring lineage-associated markers. (Figure 5B). ATI markers *PDPN* and *AGER*, as well as the intermediate marker *CDKN1A*, were highest on day 4 and declined over time, indicating that prolonged cultivation does not induce a stable ATI-like phenotype. Only *CAV1* increased notably by day 25, which may be compatible with stress-associated remodeling under prolonged high-density culture, rather than lineage commitment [22]. Basal cell markers *KRT5* and *KRT14* remained undetectable, ruling out transdifferentiation toward basal-like phenotypes.

To identify when A549 cells display the highest levels of selected ATII-associated readouts within this culture system, we analyzed the expression of ABCA3, a key transporter involved in surfactant phospholipid packaging into lamellar bodies [23]. The highest *ABCA3* transcript levels were observed on day 7, representing a 47% increase compared to day 4 (Figure 5C). In parallel, we examined the presence of surfactant protein A (SP-A), which supports surfactant assembly and function [24]. Western blot analysis revealed an approximately 40% increase in SP-A protein levels on day 7, followed by a decline and a secondary increase on day 25, reaching levels comparable to those at day 7 (Figure 5D). The temporal changes in SP-A protein abundance coincided with the accumulation of neutral lipids, whereas total cholesterol levels remained unchanged (Figure 5E). The observed increase in neutral lipids was primarily driven by elevated sterol ester content (Supplementary Figure S3A). In addition, long-term cultures (day 25) also exhibited a significant increase in free fatty acid levels (Supplementary Figure S3B), a feature commonly associated with metabolic stress and altered membrane remodeling [25,26].

Together, these data indicate that early cultivation stages (around day 7) show the highest levels of several surfactant-associated readouts measured in this study, including *ABCA3* and SP-A. At later time points, the accumulation of neutral lipids and free fatty acids, together with reduced bioenergetic activity, suggests the emergence of stress-associated metabolic remodeling under prolonged high-density culture conditions.

### 2.3. Inflammasome-Related Stress and Loss of Viability in A549 Cells Under Prolonged Static Conditions

**Figure 6:**
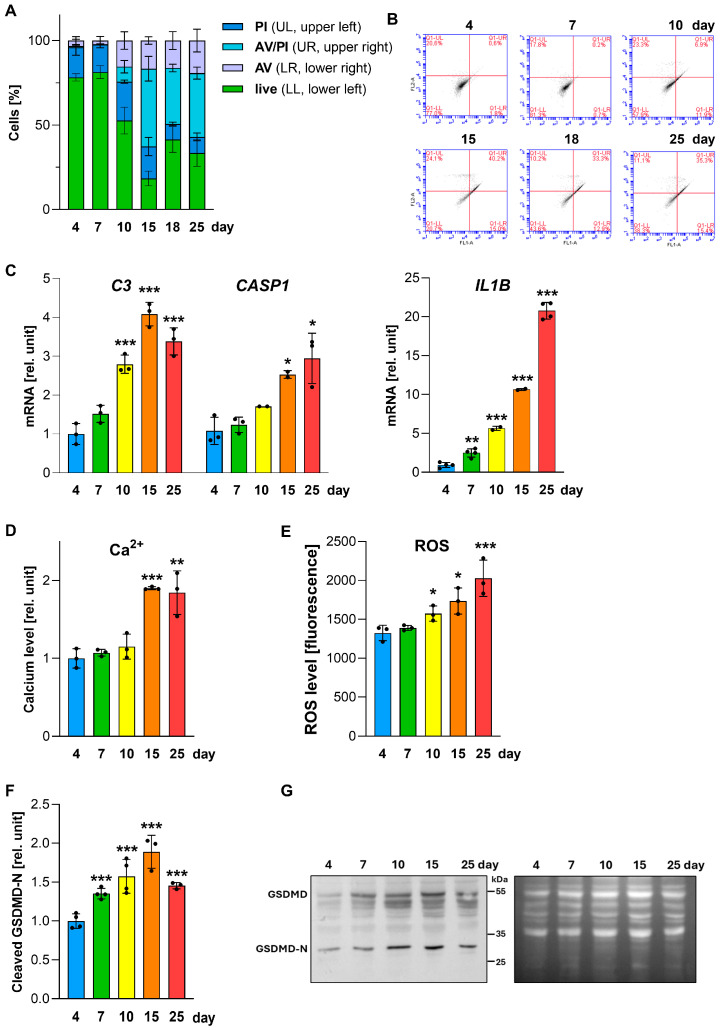
Inflammasome-associated transcripts and loss of viability in long-term cultured A549 cells. A549 cells were cultured in DMEM throughout the experiment. (**A**) Cell viability assessed by flow cytometry using Annexin V (AV) and propidium iodide (PI) staining. Data represent mean ± SD from three independent experiments. (**B**) Representative flow cytometry dot plots. (**C**) Relative transcript levels of *C3*, *CASP1*, and *IL1B* determined by RT-qPCR. (**D**) Intracellular Ca^2+^ concentration. (**E**) Reactive oxygen species (ROS) fluorescence intensity. (**F**) Relative level of cleaved gasdermin D-N (GSDMD-N). (**G**) Representative immunoblot showing full-length GSDMD and cleaved GSDMD-N (left), with No-Stain™ total-protein labeling used for normalization (right). (**C**–**F**) Data represent mean values from at least three independent experiments (individual points shown), normalized to day 4 cultures. Statistically significant differences between long-term cultures and 4-day cultures are indicated: * *p* < 0.05; ** *p* < 0.01; *** *p* < 0.001.

During long-term cultivation, A549 cells exhibited alterations in mitochondrial function, lipid metabolism, and surfactant-associated markers, including a secondary increase in SP-A protein levels at day 25. Cell viability was assessed by flow cytometry using Annexin V and propidium iodide (PI) staining. After day 10, both early apoptotic (Annexin V^+^) and late apoptotic/necrotic (Annexin V^+^/PI^+^) populations increased, with more than 35–40% of cells positive for Annexin V and/or PI at days 15 and 25 (Figure 6A,B), indicating a substantial decline in viability during prolonged static high-density culture [13,14,27].

To further characterize the nature of the observed stress responses, we analyzed the expression of genes associated with inflammasome-related inflammatory signaling. Specifically, transcript levels of *C3* (complement component 3), *CASP1* (caspase-1), and *IL1B* were significantly upregulated in cells cultured for 15 and 25 days (Figure 6C), coinciding with increased Annexin V/PI positivity. Caspase-1 is a key component of the inflammasome that processes pro-inflammatory cytokines such as IL-1β, while complement C3 also contributes to IL-1β secretion [28]. Supporting inflammasome activation, intracellular calcium levels, known to trigger this pathway, were approximately twofold higher at later time points (Figure 6D) [29,30].

Importantly, prolonged static conditions were accompanied by a progressive increase in reactive oxygen species (ROS) levels, reaching a 57% elevation at day 25 compared with day 4 (Figure 6E). Elevated ROS is a known trigger of stress-associated inflammatory signaling in multiple systems. It has been reported to promote caspase-1–dependent cleavage of Gasdermin D (GSDMD), leading to the release of its N-terminal fragment that oligomerizes within the plasma membrane and can induce pyroptotic cell death [27,31,32]. Consistent with this, an increase in the cleaved GSDMD-N fragment was observed, with the most pronounced elevation at day 15 (Figure 6F,G and Supplementary Figure S5).

Together, these data show that late time points are characterized by increased ROS/Ca^2+^ levels and upregulation of inflammasome-associated transcripts, accompanied by evidence of partial GSDMD processing. This is consistent with inflammatory stress accompanying loss of viability under prolonged static high-density culture conditions rather than demonstrating a specific programmed cell-death mechanism.

## 3. Discussion

In vitro cultures of lung epithelial cells, including A549, are widely used to study pulmonary physiology and pathology; however, their reliability strongly depends on standardized conditions, including medium composition, serum availability, and culture density/confluence [9,10,11,33]. In our study, A549 cells exhibited only minor differences in overall growth between DMEM and Ham’s F-12 media (Figure 1A). By contrast, the two media differed more clearly in intracellular phospholipid composition (Figure 2), which may in part reflect their distinct nutrient composition. Although multiple compositional differences between the media may contribute, one plausible factor is the approximately 3.5-fold higher concentration of choline chloride in Ham’s F-12 than in DMEM, which could provide greater substrate availability for PC synthesis via the choline-dependent Kennedy pathway [34]. At the same time, neither of the media used here contains ethanolamine, meaning that exogenous substrate support for PE synthesis via the CDP-ethanolamine branch of the Kennedy pathway would be absent. This difference in medium composition may therefore contribute to the distinct late-stage PC/PE balance observed under these conditions, either through relatively greater support of PC synthesis, reduced PE synthesis, or a combination of both. The most pronounced and reproducible changes were observed in lipid metabolic parameters, which were more consistently detectable in DMEM (Figure 1 and Figure 2), and this medium was therefore selected for subsequent in-depth analyses. To minimize variability arising from serum-derived lipids and acute proliferative signaling, all cultures were subjected to a uniform 48 h serum deprivation period prior to harvest, as commonly applied in lipid metabolic studies [35,36,37,38,39].

Because ATII-like cells may undergo phenotypic transitions toward other epithelial states [3,40,41], we examined marker expression during prolonged static culture. Basal epithelial markers *KRT5* and *KRT14* remained undetectable, while ATI-associated markers *PDPN* and *AGER* declined over time (Figure 5A,B), indicating that no clear transition toward a defined alternative epithelial phenotype occurred. A key finding is that A549 cells displayed the highest levels of several surfactant-associated readouts early during cultivation, peaking around day 7, as indicated by maximal *ABCA3* expression, increased SP-A abundance (Figure 5C,D), and peak lipid secretion (Figure 1B–D). This temporal window is consistent with prior studies reporting time-dependent changes in A549 phenotype and surfactant-associated functions [9,15,16,17,24]. Beyond this early phase, lipid secretion and de novo lipid synthesis progressively declined together with a marked drop in mitochondrial respiration and glycolytic capacity (Figure 4A,B), reduced abundance of respiratory complex subunits (Figure 4C–G), and decreased incorporation of [^14^C]-acetate into newly synthesized lipids (Figure 2E,F), collectively indicating suppression of anabolic metabolism. Such coordinated bioenergetic suppression is consistent with a stress-associated transition during prolonged static high-density culture. In high-density epithelial monolayers, pericellular hypoxia and broader metabolic constraint have been reported [12,13,14,21], and these factors may contribute to the transcriptional and metabolic remodeling observed at later cultivation stages.

In parallel with the metabolic decline, viability progressively decreased after approximately day 10. Flow cytometry based on Annexin V/PI double staining revealed a progressive increase in Annexin V-positive cells and a marked rise in Annexin V^+^/PI^+^ double-positive populations (Figure 6A,B), indicating increasing loss of plasma membrane integrity and viability under prolonged high-density conditions [12,14,21]. While this pattern can be compatible with late-stage apoptosis/secondary necrosis, the concurrent upregulation of *CASP1*, *IL1B*, and *C3* transcripts (Figure 6C) is consistent with inflammasome-related and pyroptosis-associated signalling [42,43,44]. This interpretation is supported by the elevation in intracellular Ca^2+^ (Figure 6D), a recognized trigger of inflammasome activation [30]. In line with this, an increase in the cleaved GSDMD-N fragment was observed (Figure 6F). GSDMD pore formation has been shown to promote membrane permeabilization and ion fluxes, including Ca^2+^ influx, in inflammasome-linked settings [45,46]. Such membrane permeabilization provides a plausible route for PI entry, consistent with the increased Annexin V^+^/PI^+^ positivity observed at later time points (Figure 6A,B) and with flow-cytometric readouts commonly reported in pyroptosis-oriented studies [47,48]. This is further supported by elevated ROS levels at later time points (Figure 6E), which have been reported to amplify inflammasome/pyroptosis-associated signalling in inflammatory contexts [32]. Previous studies in A549 cells further showed that inflammatory stimulation can increase SP-A expression, suggesting that the secondary rise in SP-A observed at day 25 (Figure 5D) could, at least in part, reflect stress- or inflammation-associated regulation in late cultures [49,50,51]. At the same time, the combination of increased ROS, elevated intracellular Ca^2+^, neutral-lipid accumulation, metabolic decline, and reduced viability is also compatible with a senescence-like stress phenotype [52,53,54,55]. Thus, our data likely reflect an early phase of maximal surfactant-associated activity, followed by a later stress-associated state that includes features consistent with both inflammasome-related signalling and senescence-like remodeling [56,57].

Two additional factors relevant to prolonged static cultures are antibiotic exposure and transient serum withdrawal. Both penicillin–streptomycin supplementation and serum deprivation have been reported to modulate gene expression, metabolic activity, and cellular stress responses in cultured cells, even when applied at standard concentrations and durations [58,59,60,61]. While these conditions were applied uniformly across all time points in this study, their physiological impact may be amplified in late-stage cultures with reduced metabolic reserve, potentially contributing to the stress-associated phenotype observed at later stages.

Reduced de novo phospholipid synthesis was accompanied by intracellular accumulation of free fatty acids, triacylglycerols, and sterol esters (Figure 5E; Supplementary Figure S3). Such lipid remodeling is consistent with compensatory storage responses to oxidative and metabolic stress [62,63,64]. The pronounced decline in cellular PG levels after day 10 further indicates a reduction in the pool of PG available for secretion within this culture system, which may contribute to the observed decrease in surfactant-associated lipid output [65,66]. Together, these results identify a temporal window around day 7 during which static A549 cultures exhibit the highest levels of surfactant-associated metabolic readouts measured in this study. At later stages, the system is increasingly characterized by suppressed bioenergetic capacity, altered lipid handling, inflammatory stress signatures, and reduced viability. These observations therefore define the behavior and limitations of A549 cells under prolonged static culture. Although A549 cells retain selected surfactant-associated features, they do not fully recapitulate native ATII cell biology, as previous studies have shown that the molecular species composition of secreted PG and PC differs from that found in primary human ATII cells [67,68,69,70]. Their tumor origin likely further contributes to the metabolic plasticity, altered differentiation behavior, and stress responses observed here. Primary ATII cells or other non-tumor alveolar epithelial models may therefore be more appropriate for studies specifically focused on physiological surfactant homeostasis. Prolonged static cultivation would, however, also be expected to impose density- and nutrient-related constraints in such systems, although their magnitude and kinetics may differ from those observed in A549 cells. At the same time, A549 cells remain a practical and experimentally accessible model for selected surfactant-associated and metabolic studies, provided that their temporal phenotypic drift and model-specific limitations are taken into account. These findings emphasize that both cultivation duration and the accompanying increase in culture density should be explicitly considered when interpreting long-term static A549 experiments, particularly in studies aiming to investigate lipid metabolism or surfactant-related cellular processes in this widely used in vitro model.

## 4. Materials and Methods

### 4.1. Mammalian Cell Culture

A549 human alveolar basal epithelial cells (CCL-185, ATCC, Manassas, VA, USA), originally derived from a human lung adenocarcinoma [71], were cultured in either low-glucose Ham’s F12 (N3520, Sigma-Aldrich, St. Louis, MO, USA) or low-glucose DMEM (LM-D1099, Biosera, Nuaille, France), supplemented with 10% fetal bovine serum (FBS, F7524, Sigma-Aldrich, St. Louis, MO, USA) and 100 U/mL penicillin/streptomycin (XC-A4122, Biosera, Nuaille, France). Cells were maintained at 37 °C in a humidified incubator with 5% CO_2_.

For experiments spanning 4–25 days, parallel long-term cultures were established in culture vessels appropriate for the respective downstream assays: (i) 90 mm tissue-culture dishes were seeded at 2 × 10^5^ cells per dish and maintained under standard culture conditions for biochemical assays requiring bulk material (protein, RNA, lipid extractions); (ii) 96-well tissue-culture plates were seeded at 1–2 × 10^3^ cells per well for plate-reader–based assays (Seahorse measurement, ROS, Ca^2+^). Edge wells were filled with sterile phosphate-buffered saline to minimize evaporation during prolonged cultivation.

Medium was refreshed every 2–4 days. Cultures were maintained as static (non-passaged) monolayers for the indicated durations. Forty-eight hours before sample collection, cultures were switched to serum-free medium to minimize interference from exogenous serum lipids. This serum-free period was applied uniformly across all time points. Total cellular protein (Bradford assay; Bio-Rad, Hercules, CA, USA) was recorded per dish or per well to document culture expansion (cell accumulation), and for normalization of downstream assays where indicated, consistent with the common use of total protein for normalization to differences in cell number/cell accumulation in cell-based workflows [72,73]. As an exception, for Seahorse XF measurements, protein content was determined using the DC Protein Assay (Bio-Rad, Hercules, CA, USA).

### 4.2. Radioactive Labeling of Lipids

A549 cells were incubated with [^14^C]-acetate (0.5 µCi/mL) for 48 h prior to sample collection to label newly synthesized lipids. After labeling, cell pellets and 900 µL of the corresponding culture medium were collected for lipid extraction. Lipids were extracted using chloroform:methanol:HCl (60:30:0.26, *v*/*v*/*v*) and shaken for 1 h. Then, 0.1 M MgCl_2_ was added, the mixture was vortexed, and incubated for 30 min to remove residual contaminants. Phases were separated by centrifugation (10 min, 460× *g*), and the organic phase was collected.

For cell-associated lipids, individual lipids were separated by thin-layer chromatography (TLC) on Silica Gel 60 plates (Sigma-Aldrich, St. Louis, MO, USA) using chloroform:methanol:acetic acid (65:25:8, *v*/*v*/*v*). The TLC plates were dried and exposed to a phosphorimaging screen for 1 week. Radioactive signals were detected using a Bio-Rad scanner, and relative percentages of individual phospholipids were quantified with Quantity One software (version 4.5.2, Bio-Rad, Hercules, CA, USA).

For medium-associated lipids, the organic phase was concentrated under a nitrogen stream, mixed with Bray scintillation cocktail, and radioactivity was measured using a Beckman LS-6000-TA Liquid Scintillation Counter (Beckman Coulter, Brea, CA, USA). Data were normalized to mL of medium and mg of total cellular protein.

### 4.3. Determination of Inorganic Phosphate in Culture Medium After Lipid Extraction

A549 cells were cultured for 4, 7, 10, 15, or 25 days. Culture medium was collected, and lipids were extracted as previously described using chloroform:methanol:HCl (60:30:0.26, *v*/*v*/*v*). Inorganic phosphate (P_i_) was quantified using a 1-amino-2-naphthol-4-sulfonic acid (ANSA)–molybdate colorimetric assay after acid digestion, following the laboratory protocol described in [74]. Briefly, to the concentrated lipid fraction, 200 µL of a sulfuric acid:perchloric acid mixture (9:1, *v*/*v*) was added, and samples were heated in a sand bath at 180 °C for 30 min. After cooling to room temperature, 4.8 mL of ANSA-molybdate reagent was added, and samples were incubated for 30 min at 105 °C. Samples were cooled to room temperature and vortexed. Subsequently, 200 µL of each sample was transferred to a 96-well plate, and absorbance was measured at 830 nm. P_i_ levels were determined using a calibration curve prepared from K_2_HPO_4_ standards. Phosphate content was normalized to total cellular protein and mL of medium (µg P_i_/mL medium/mg protein).

### 4.4. Targeted Lipidomics Analysis

A549 cells were cultured in DMEM for 4, 7, 10, 15, and 25 days. Two days prior to medium collection, FBS-containing medium was replaced with FBS-free medium. Collected medium was centrifuged (5 min, 460× *g*) and stored at –20 °C.

For the extraction of lipids, cell medium samples were thawed on ice and vortexed, and 100 µL was transferred to a new 2 mL tube. A total of 10 µL of SPLASH Lipidomix internal standard mixture was added, and the samples were vortexed and incubated on ice for 15 min. A blank sample was prepared following the same procedure as the study samples, where ultrapure water was used instead of the biological sample and no internal standards were added. Lipids were extracted using a two-phase methyl-tert-butyl ether (MTBE)/methanol extraction as described in [75]. Briefly, 375 µL cold methanol was added, followed by 1.250 µL MTBE, vortexing after each addition. Samples were incubated for 1 h at 4 °C on an orbital shaker (32 rpm). Phase separation was induced by adding 375 µL H_2_O, followed by vortexing and 10 min incubation at 4 °C on the orbital shaker. Samples were centrifuged (10 min, 4 °C, 1000× *g*), and the upper organic phase (1 mL) was collected into a new 2 mL tube. Extracts were evaporated under vacuum using a Christ RVC 2-25 CD plus rotary vacuum concentrator ((Martin Christ Gefriertrocknungsanlagen GmbH, Osterode am Harz, Germany) at room temperature and 70 mbar.

The dried extracts were immediately stored at −80 °C. Before the lipidomics analysis, the dried extracts were reconstituted in 100 µL of 100% isopropanol and vortexed thoroughly (15 s). An aliquot (10 µL) of each extract was pooled into a quality control sample, which was used for the monitoring of the signal stability of the mass spectrometer. A targeted lipidomics method, previously used for clinical studies on various matrices such as serum, plasma, cerebrospinal fluid, brain tissue, sweat [76,77,78], and more, was used to semiquantify lipids in the cell medium extracts. The liquid chromatography (Sciex ExionLC system (SCIEX, Framingham, MA, USA) with Waters Acquity BEH C8 2.1 mm, 100 mm, 1.7 μm column (Waters Corporation, Milford, MA, USA)) and mass spectrometry (Sciex QTrap 6500+, SCIEX, Framingham, MA, USA) parameters and settings followed our previously published methodology [76,77,78]. The analysis was controlled by the Analyst software (version 1.7.2, SCIEX, Framingham, MA, USA), and the data were processed by SCIEX OS software (version 1.6.1, SCIEX, Framingham, MA, USA). Finally, the data were normalized to mg of cellular protein and mL of culture medium.

### 4.5. Neutral Lipid Quantification

Neutral lipids were separated by TLC and visualized by sulfuric-acid charring, as detailed in the laboratory workflow described in [79]. Following the sulphuric acid stain, relative lipid content was determined using CAMAG WinCATS software (version 1.4.6, CAMAG, Muttenz, Switzerland) after scanning TLC plates on CAMAG TLC scanner 3 (CAMAG, Muttenz, Switzerland) at 475 nm.

### 4.6. PGS1 Activity

PGS1 activity was measured using a radiometric incorporation assay (cytidine diphosphate–diacylglycerol (CDP-DAG) and [^14^C]glycerol-3-phosphate), according to the laboratory protocol described in [80]. The assay was performed in the presence of 50 mM MES-HCl, pH 7.0, 0.1 mM MnCl_2_, 0.083 mM CDP-DAG, 1 mM Triton X-100, 0.02 mM [^14^C]glycerol-3-phosphate (40,000 CPM/nmol), and whole-cell homogenates corresponding to 75 μg of proteins in a total volume of 120 μL for 60 min at 37 °C. The reaction was stopped by the addition of 1.5 mL chloroform:methanol:HCl mixture (100:100:0.6, *v*/*v*/*v*), followed by the addition of 1 mL of water to produce two phases. Aliquots of 0.45 mL of the organic layer were transferred into new vials. After evaporation of the organic phase, lipids were separated on Silica gel 60 TLC plates using chloroform:methanol:acetic acid: water developing solvent (75:45:3:1, *v*/*v*/*v*), visualized by phosphorimaging and quantified by Quantity One software (BioRad, Hercules, CA, USA).

### 4.7. RNA Extraction and Quantitative Real-Time PCR

Total RNA was isolated using the GeneJET RNA Purification Kit (Thermo Scientific, Waltham, MA, USA). One microgram of RNA per sample was reverse-transcribed with the LunaScript^®^ RT SuperMix Kit (New England Biolabs, Ipswich, MA, USA). Quantitative real-time PCR was performed on an AriaMx Real-Time PCR System (Agilent Technologies, Santa Clara, CA, USA) using the LightCycler 480 SYBR Green I Master mix (Roche, Basel, Switzerland). Gene expression levels were normalized to *ACTB* and *GAPDH*. Relative expression was calculated using the 2^−ΔΔCt^ method. Primer sequences are listed in Supplementary Table S1.

### 4.8. Western Blot Analysis

Proteins were extracted from cell homogenates, separated on 12% SDS-PAGE gels, and transferred onto nitrocellulose membranes. Membranes were blocked overnight with 5% milk in Tris-buffered saline (50 mM Tris-HCl, pH 8.0; 150 mM NaCl). Primary antibodies used were: rabbit anti-SP-A (AB3420-I, 1:1000; Sigma-Aldrich, St. Louis, MO, USA), rabbit anti-GSDMD (126–128) antibody (G7422, 1:1000, Sigma-Aldrich, St. Louis, MO, USA), mouse anti-β-Actin (8H10D10, 1:3300; Abcam, Cambridge, UK), and mouse OxPhos Human WB Antibody Cocktail (1:500; Thermo Fisher Scientific, Waltham, MA, USA). Horseradish peroxidase-conjugated secondary antibodies (anti-mouse or anti-rabbit, 1:30,000; Sigma-Aldrich, St. Louis, MO, USA) were visualized with SuperSignal™ West Pico PLUS Chemiluminescent Substrate (Thermo Scientific, Waltham, MA, USA). Band intensities were quantified by densitometry. Depending on the experiment, normalization was performed either to the corresponding total-protein signal per lane obtained with No-Stain™ labeling (Thermo Scientific, Waltham, MA, USA) or to β-actin, as indicated in the corresponding figure legends.

### 4.9. Seahorse Mitochondrial Assay

A total of 1–2 × 10^3^ A549 cells were seeded per well in a Seahorse XF96 cell culture microplate (Agilent Technologies, Santa Clara, CA, USA) and cultured for 4, 7, 10, 15 and 25 days. Medium was refreshed every 2–4 days as described above. Medium was replaced with Seahorse XF DMEM (pH 7.4) supplemented with 10 mM glucose, 2 mM L-glutamine and 1 mM pyruvate and cells were incubated for 1 h at 37 °C. Oxygen consumption rate was measured using the Seahorse XF ProAnalyzer (Agilent Technologies, Santa Clara, CA, USA). Mitochondrial modulators were used at the following concentrations: 1.25 µM oligomycin, 1 µM FCCP, and 1 µM rotenone + 1.25 µM antimycin A. Protein content in each well was measured post-assay by DC protein assay (BioRad, Hercules, CA, USA) for basal respiration and ECAR normalization.

### 4.10. Detection of Cell Viability by Flow Cytometry Assay

Apoptosis and necrosis were assessed using double staining with Annexin V and PI. A549 cells cultured for 4–25 days were harvested and transferred to microtubes. The tubes were then centrifuged for 5 min at 550× *g* and at a temperature of 20 °C. The pellet was washed once with serum-free DMEM medium and centrifuged under identical conditions. The resulting pellet was resuspended in binding buffer (10 mM HEPES/NaOH, pH 7.4, 140 mM NaCl, 5 mM CaCl_2_), followed by the addition of Annexin V FLUOS (final concentration 0.5 µg/mL). The samples were incubated for 15 min at room temperature in the dark. Immediately prior to flow cytometric analysis, PI was added to the sample (final concentration 10 µM). Fluorescence signals were acquired using an Accuri C6 flow cytometer (BD Biosciences, San Jose, CA, USA). The apoptotic populations were classified as either early apoptotic (Annexin V^+^/PI^−^) or late apoptotic/necrotic (Annexin V^+^/PI^+^), and the percentage of each population was quantified at each time point.

### 4.11. Calcium Detection with Fluo-3-Acetoxymethyl (Fluo-3AM)

A549 cells cultured for up to 25 days were loaded with 3 µM Fluo-3AM (AAT Bioquest, Sunnyvale, CA, USA) and 2 mM probenecid for 45 min at 37 °C. Excess dye was removed by washing with Cl^−^ wash buffer (pH 7.4; 130 mM NaCl, 5 mM KCl, 10 mM Na-Hepes, 2 mM CaCl_2_, 10 mM glucose, 2 mM probenecid). Fluorescence was measured at excitation 490 nm and emission 525 nm. Intracellular calcium levels were normalized to protein content determined by Bradford assay.

### 4.12. ROS Detection

Intracellular ROS levels in A549 cells were measured using the ROS/Superoxide Detection Assay Kit (Cell-based, ab139476, Abcam, Cambridge, UK) according to the manufacturer’s instructions. Briefly, cells were cultured for the indicated time points, washed with phosphate-buffered saline, and incubated with the ROS/Superoxide detection reagent for 45 min at 37 °C protected from light. Following incubation, cells were washed, and fluorescence was measured using a fluorescence microplate reader (excitation/emission: 490/525 nm). Fluorescence signals were normalized to total cellular protein.

### 4.13. Statistical Analysis

Statistical comparisons were carried out by one-way analysis of variance using SigmaPlot 14 software (Systat Software, San Jose, CA, USA). All graphs (GraphPad Prism 10 software, GraphPad Software, San Diego, CA, USA) show the mean ± SD. Where appropriate, post hoc multiple comparison testing was performed (Holm–Sidak). The number of independent experiments (biological replicates) is stated in the corresponding figure legends (typically ≥3).

## 5. Conclusions

In summary, prolonged static cultivation of A549 cells leads to progressive metabolic and bioenergetic decline, including reduced PG biosynthesis and increased oxidative stress. These changes coincide with reduced viability and increased expression of inflammasome-associated transcripts. Our results identify an early cultivation window (approximately up to day 7) during which A549 cultures display the highest levels of surfactant-associated lipid secretory activity, whereas later stages are dominated by a stress-associated high-density phenotype.

## Data Availability

The original contributions presented in this study are included in the article/Supplementary Material. Further inquiries can be directed to the corresponding author.

## Supplementary Materials

The following supporting information can be downloaded at: https://www.mdpi.com/article/10.3390/ijms27083417/s1. Reference [81] is cited in the Supplementary Material.
