# Supplementary material for: Long-Term Static Cultivation Alters Lipid Metabolism and Bioenergetic Capacity in A549 Cells
Source: Int J Mol Sci. 2026 Apr 10;27(8):3417. doi: 10.3390/ijms27083417 (PMC13116223; doi:10.3390/ijms27083417)
Supplement: Supplementary file 1 [file ijms-27-03417-s001.zip › ijms-4219824-supplementary.pdf]

SUPPLEMENTAL TABLE AND FIGURES

Supplementary Table S1. List of primers.

Name of primer	Sequence	Source
ACT_for	ATTGGCAATGAGCGGTTCCG	[81]
ACT_rev	AGGGCAGTGATCTCCTTCTG	
GADPH_ for	CACCGTCAAGGCTGAGAAC	www.origene.com
GADPH_ rev	GAGGGATCTCGCTCCTGG	
C3_ for	GTGGAAATCCGAGCCGTTCTCT	www.origene.com
C3_ rev	GATGGTTACGGTCTGCTGGTGA	
CASP1_ for	GCTGAGGTTGACATCACAGGCA	www.origene.com
CASP1_ rev	TGCTGTCAGAGGTCTTGTGCTC	
IL1β_ for	CCACAGACCTTCCAGGAGAATG	www.origene.com
IL1β_ rev	GTGCAGTTCAGTGATCGTACAGG	
KI67_ for	GAAAGAGTGGCAACCTGCCTTC	www.origene.com
KI67_ rev	GCACCAAGTTTTACTACATCTGCC	
PCNA_ for	CAAGTAATGTCGATAAAGAGGAGG	www.origene.com
PCNA_ rev	GTGTCACCGTTGAAGAGAGTGG	
CAV1_ for	TCTTCCTTCCTCAGTTCCCT	[41]
CAV1_ rev	GTAGAGATGTCCCTCCGAGTC	
PDPN_ for	GTGCCGAAGATGATGTGGTGAC	[41]
PDPN_ rev	GGACTGTGCTTTCTGAAGTTGGC	
AGER_ for	GCTGTCAGCATCAGCATCAT	[41]
AGER_ rev	ATTCAGTTCTGCACGCTCCT	
CDKN1A_ for	CCAGCATGACAGATTTCTACCA	[41]
CDKN1A_ rev	GAACCTCTCATTCAACCGCC	
ABCA3_ for	CCTACCTCATCCTGCTCTTCC	[41]
ABCA3_ rev	GTATAATTCTGTCAGTGTCCGCC	

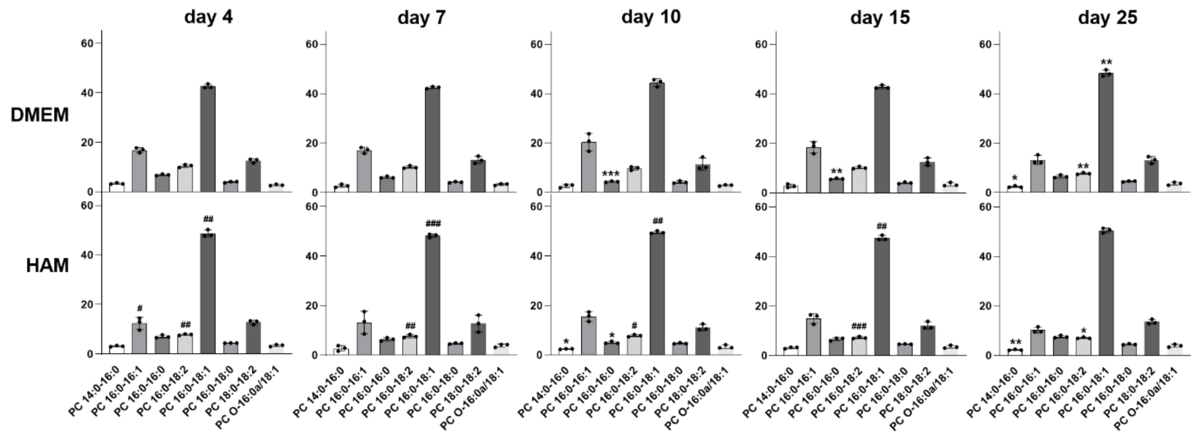

Supplementary Figure S1. Fatty acyl chain composition of secreted phosphatidylcholine. A549 cells were cultured for 4, 7, 10, 15, or 25 days in low-glucose DMEM or Ham's F-12 medium. Culture supernatants were collected, centrifuged, and analyzed by mass spectrometry to determine the fatty acyl chain composition of phosphatidylcholine (PC), as described in Materials and Methods. Data represent mean ± SD from three independent experiments (individual points shown). Statistically significant differences between prolonged cultures and 4-day cultures in the respective medium (asterisks) or between Ham's F-12 and DMEM (hashtags) are indicated: * or # $p < 0.05$; ** or ## $p < 0.01$; *** or ### $p < 0.001$. PC, phosphatidylcholine

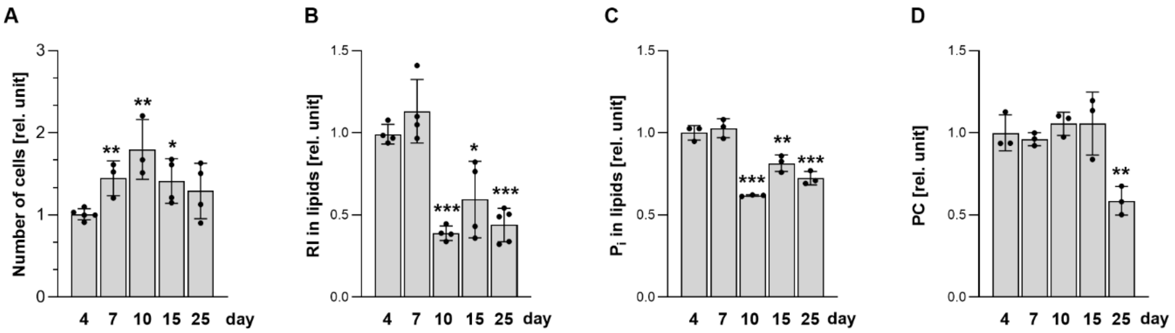

Supplementary Figure S2. Lipid secretion in A549 cells normalized to cell number.

A549 cells were cultured for 4, 7, 10, 15, or 25 days in low-glucose DMEM medium. Culture supernatants were collected, centrifuged, and analyzed by mass spectrometry to determine the fatty acyl chain composition of phosphatidylcholine, as described in Materials and Methods. (A) Secretion of total lipids into the culture medium, assessed using $[^{14}C]$-acetate labeling. (B) Secretion of phospholipids and sphingolipids into the medium, quantified based on inorganic phosphate content (P_i). (C) Secretion of phosphatidylcholine (PC) into the medium, measured by mass spectrometry. (A–C) Lipid amounts were normalized to cell number. Data represent mean ± SD from at least three independent experiments (individual points shown). Statistically significant differences between long-term cultures and 4-day cultures are indicated by asterisks: $*p < 0.05$; $**p < 0.01$; $***p < 0.001$. RI, radioactivity

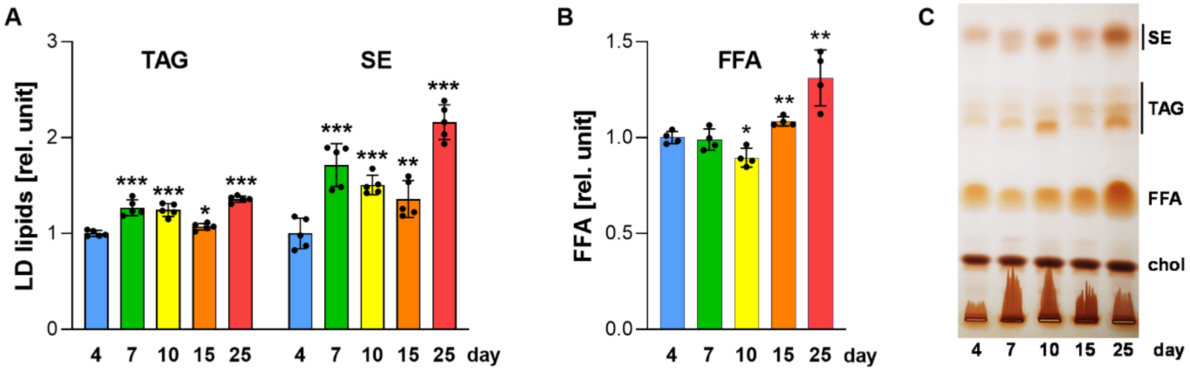

Supplementary Figure S3. Levels of neutral lipids in A549 cells during prolonged culture. A549 cells were cultured for 4, 7, 10, 15, or 25 days in low-glucose DMEM or Ham's F-12 medium. Neutral lipids were extracted and quantified as described in Materials and Methods. (A) Relative levels of triacylglycerols (TAG) and sterol esters (SE) and (B) free fatty acids were determined and normalized to 4-day cultures. Data represent mean ± SD from at least four independent experiments (individual points shown). (C) Representative TLC image showing separation of neutral lipids. Statistically significant differences compared to 4-day cultures are indicated: *$p < 0.05$; **$p < 0.01$; ***$p < 0.001$. Chol, cholesterol; FFA, free fatty acids; LD, lipid droplet; SE, sterol esters; TAG, triacylglycerols.

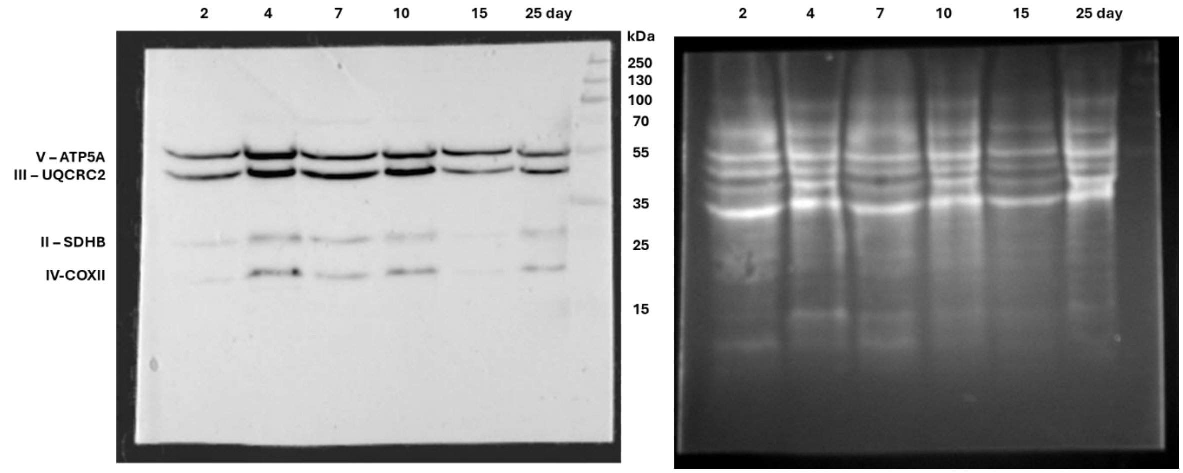

Supplementary Figure S4. Full-length, uncropped blots corresponding to Figure 4C. Representative immunoblot showing protein levels of mitochondrial respiratory chain subunits SDHB (Complex II, II), UQCRC2 (Complex III, III), COXII (Complex IV, IV), and ATP5A (ATP synthase, V) (left). Total protein staining used for normalization is shown on the right and was performed using the No-Stain™ Protein Labeling Reagent (A44449). Immunoblotting was carried out using the OxPhos Human WB Antibody Cocktail (1:500; Invitrogen).

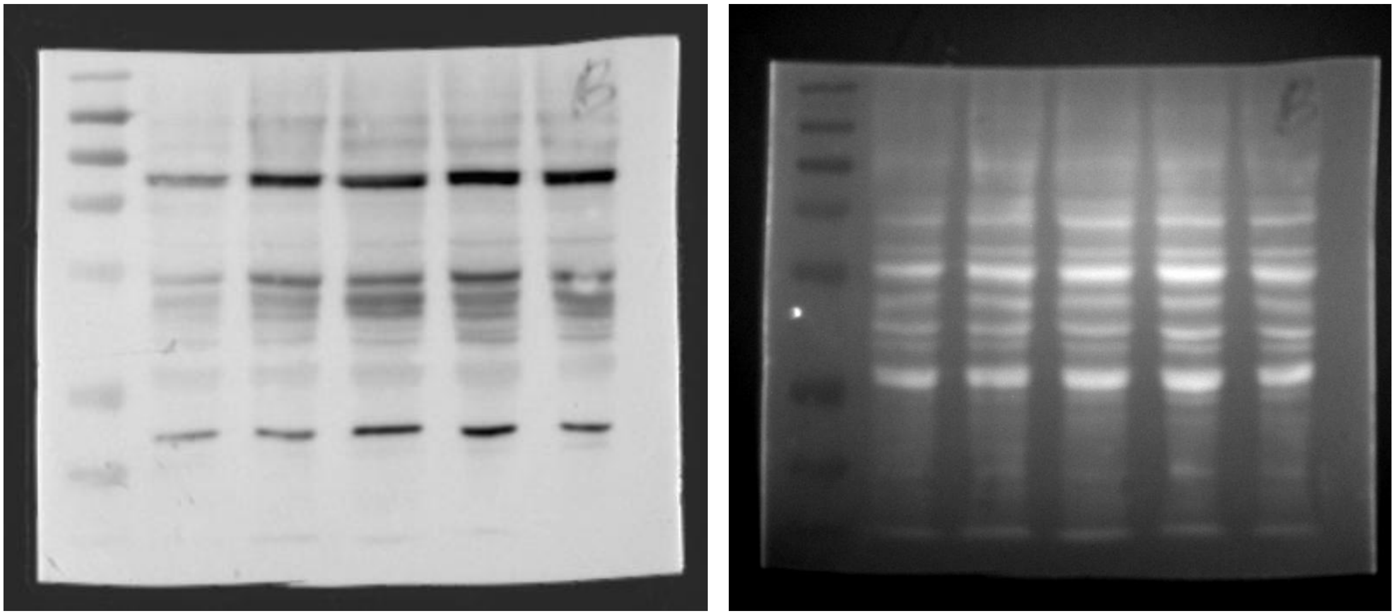

Supplementary Figure S5. Full-length, uncropped blots corresponding to Figure 6G. Representative immunoblot showing full-length GSDMD and cleaved GSDMD-N (left), with No-Stain™ total-protein labeling used for normalization (right). Total protein staining used for normalization is shown on the right and was performed using the No-Stain™ Protein Labeling Reagent (A44449). Immunoblotting was carried out using the rabbit anti-GSDMD antibody (G7422, 1:1000, Sigma-Aldrich)
